# Context of a neonatal death affects parental perception of end-of-life care, anxiety and depression in the first year of bereavement

**DOI:** 10.1186/s12904-023-01183-8

**Published:** 2023-05-13

**Authors:** Gilles Cambonie, Chloé Desage, Pénélope Thaller, Anne Lemaitre, Karine Bertran de Balanda, Clémentine Combes, Arthur Gavotto

**Affiliations:** 1 Department of Neonatal Medicine and Paediatric Intensive Care Unit, Arnaud de Villeneuve Hospital, Montpellier University Hospital Centre, University of Montpellier, 371 Avenue du Doyen Gaston Giraud, 34295 Cedex 5 Montpellier, France; 2 Pathogenesis and Control of Chronic Infection, UMR 1058, INSERM, University of Montpellier, Montpellier, France; 3 PhyMedExp, CNRS, INSERM, University of Montpellier, Montpellier, France

**Keywords:** Anxiety, Depression, End-of-life decisions, Mortality, Newborn, Parental grief, Withholding or withdrawing care

## Abstract

**Background:**

Neonatal death is often preceded by end-of-life medical decisions. This study aimed to determine whether the context of death − after a decision of withholding or withdrawing life-sustaining treatment (WWLST) or despite maximum care − was associated with subsequent risk of parental anxiety or depression. The secondary objective was to assess parents’ perceptions of end-of-life care according to death context.

**Methods:**

Prospective single center observational study of all neonatal deaths in a neonatal intensive care unit over a 5-year period. Data were collected during hospitalization and from face-to-face interviews with parents 3 months after the infant’s death. Anxiety and depression were assessed using Hospital Anxiety and Depression Scale (HADS) questionnaires, completed by parents 5 and 15 months after death.

**Results:**

Of 179 deaths, 115 (64%) occurred after the WWLST decision and 64 (36%) despite maximum care. Parental satisfaction with newborn care and received support by professionals and relatives was higher in the first condition. Sixty-one percent of parents (109/179) attended the 3-month interview, with the distribution between groups very close to that of hospitalization. The completion rates of the HADS questionnaires by the parents who attended the 3-month interview were 75% (82/109) at 5 months and 65% (71/109) at 15 months. HADS scores at 5 months were consistent with anxiety in at least one parent in 73% (60/82) of cases and with depression in 50% (41/82). At 15 months, these rates were, respectively, 63% (45/71) and 28% (20/71). Risk of depression at 5 months was lower after a WWLST decision (OR 0.35 [0.14, 0.88], *p* = 0.02). Explicit parental agreement with the WWLST decision had an equivocal impact on the risk of anxiety at 5 months, being higher when expressed during hospitalization, but not at the 3-month interview.

**Conclusions:**

Context of death has a significant impact on the emotional experience of parents after neonatal loss, which underlines the importance of systematic follow-up conversations with bereaved parents.

**Supplementary Information:**

The online version contains supplementary material available at 10.1186/s12904-023-01183-8.

## Background

The loss of a loved one is one of the most traumatic events in human life, and the intensity of grief is even higher for parents who have lost a child. Increased risk of anxiety, depression, somatic disease, and mortality have been reported among bereaved parents [1–3].

Neonatal death arouses feelings of profound sadness, shame, guilt and anger in parents [4]. Compassionate, individualized, and skilled support from health professionals is currently part of the standard of care [5]. Although testimonies underline the importance of this emotional sharing between parents and healthcare teams [6], investigations into the experiences and feelings of parents are still needed to achieve a deeper understanding of their emotions and needs and to enable more adequate care, information and support during hospitalization and after the infant’s death.

Neonatal death in high-income countries is often preceded by end-of-life medical decisions [7, 8]. A study conducted in the department of neonatal medicine of our institution showed that half the deaths occurred in this context [9]. The review of our practices indicated that they were in most cases in line with the ethical and legal framework in our country [10], however it did not assess how parents and caregivers felt about the decision to limit or withdraw treatment. Few data are available on how the information given to parents and their contribution to the decision of withholding or withdrawing life-sustaining treatment (WWLST) impact psychological well-being [11, 12].

The main objective of this study was to determine whether the context of a neonatal death − after a WWLST decision or despite maximum care − was associated with the subsequent risk of parental anxiety or depression. The secondary objectives were to describe the opinions of caregivers, physicians and parents on end-of-life care and information.

## Methods

This prospective observational study was performed in the department of neonatal medicine of the University Hospital of Montpellier (France). This department has a capacity of 47 beds, including 14 type 3 beds, corresponding to a neonatal intensive care unit (NICU), 18 type 2b beds, and 15 type 2a beds. The difference between bed types relates to patient’s clinical severity, which establishes a ratio between the number of patients and the number of nurses for each bed type. The number of nurses per neonate is framed by the French perinatal decrees of 1998 – i.e., one nurse for two neonates for type 3 beds, one nurse for three neonates for type 2b beds, and one nurse for six neonates for type type 2a beds. The medical team is made up of 10 senior pediatricians and 3 fellows. The median (interquartile range) annual number of births in the center during the recruitment period was 3392 (3336–3560), including 14.7 (14.3–15.0) % preterm deliveries.

The physician regularly involved in the care of a particular infant is named the referent physician or practitioner. According to the French law [10], the referent physician triggers the organization of the multidisciplinary ethics meeting, if the patient's clinical situation requires it. The referent physician presents the case during the meeting and requests the committee's reflection on withholding or withdrawing life sustaining treatments. The referent physician records the decisions taken and the reasons for them in the child's medical file. This physician also receives the parents at the end of the meeting and engage with them a discussion on the decisions taken by the committee. In the context of the study, the referent physician collected parental agreement with these decisions after ethics debate.

Caregivers inform the parents that they can make an appointment with one of the psychologists working in the department, described as referent for the family. Two psychologists work in the department and distribute the families according to their availability. Thus, each family has a referent psychologist, whether or not the parents accept appointments during or after hospitalization. This referent psychologist is present at the multidisciplinary ethics meeting and the interview 3 months after infant death. In the context of the study, the referent psychologist collected and analyzed, with the principal investigator, information during the interview with parents 3 months after infant’s death.

### Population

Parents were included if they met the following conditions: (i) death of their newborn during the study period and (ii) signature of the study participation agreement. This study was not proposed to parents if (i) the deceased infant was > 28 days of age at admission to the department, if born full term; (ii) the deceased infant had a corrected gestational age > 41 weeks at admission to the department, if born preterm; and (iii) they could not read French.

### Study period

The recruitment period included the deaths of newborns admitted to the department from January 2011 to December 2015. The study ended at the deadline for receiving the last parental questionnaires, i.e., 31 July 2017 for children who died during the first trimester of 2016.

### Data collection

Data were collected in a case report form (Supplementary file) completed at various stages (see below) by (i) the referent physician and the referent psychologist after the multidisciplinary ethics meeting; (ii) the physician and the nurse present at the moment of the infant’s death; (iii) the principal investigator and the referent psychologist after the interview with the parents 3 months after the death of their infant; and (iv) the principal investigator, upon receipt of the Hospital Anxiety and Depression Scale (HADS) questionnaires sent by the parents.

#### Multidisciplinary ethics meetings

These meetings were considered when providing care appeared as “unreasonable obstinacy” taking into account life expectancy or the expected quality of life. Unreasonable obstinacy is defined by the implementation or maintenance of treatments which appear useless and disproportionate or which have no other effect than the sole artificial maintenance of life [10]. Information was gathered on the reason for the meeting, the infant's main somatic features (perinatal characteristics, postnatal age, respiratory and hemodynamic support, and the main organ failures), the meeting participants, and the decision made at the conclusion of the meeting. The parents were informed beforehand of this meeting, and that feedback on the committee's opinion would be provided. They were received for an interview by the referent physician and the nurse immediately after the multidisciplinary ethics meeting. The limitations and/or withdrawal of care were clearly stated and their opinions were sought. The referent physician and the nurse then classified the parental opinions about the decision as explicit agreement, tacit agreement, ie, parents whose attitude or expression suggested that they did not oppose the decision, disagreement, or impossible/difficult to adjudicate.

#### Death

The information mainly focused on parental presence, the infant’s pain or discomfort, and support provided to the family.

#### Interview 3 months after infant death

A face-to-face interview with the head of department, i.e., the principal investigator, and the referent psychologist was systematically offered. The interview was announced at the time of death, then a letter was sent to the parents 6–8 weeks after the death, providing the date and place of the meeting, located in the office of the referent psychologist, ie, 2 floors above from the department of neonatal medicine. The letter mentioned that the interview would focus on reviewing the medical history of their child, answering their questions, and providing a better understanding of the events that occurred during the hospitalization. It was also specified that particular attention would be paid to the family experience related to the loss of the infant.

In practice, this interview proceeded through four steps: (i) presentation of the objectives of the meeting; (ii) expression of the parents' feelings and answers to their questions; and (iii) a semi-structured interview on the family environment and the experience of the infant’s hospitalization and death and the parents' current feelings and family reorganization. The last part of the interview was devoted to (iv) a detailed presentation of the study.

Family environment was assessed from hospitalization, during medico-psycho-social meetings -which gather each week the medical team, nurses, psychologists and social workers of the department- and appointments between the referent psychologist and the parents. In addition, some information was provided by the parents during the interview 3 months after infant death. The main environmental factors collected were understanding and expression of French, communication within the couple, support from relatives, social vulnerability (mainly assessed from the financial difficulties for the everyday life, including transport or the cost of accommodation close to hospital), family vulnerability (mainly assessed from the relationships and support from their own parents and siblings), psychological vulnerability (mainly assessed form a past-history of bereavement and the requirement of psychotropic medication for various causes). Considering the different sources of information for family environment, the assessment was based on the joint opinion of the principal investigator and the referent psychologist. For the purpose of standardization, this joint opinion was summarized on a 5-point scale. Satisfactory ratings (for French understanding and expression, communication within the couple, and support from relatives) and presence of a significant vulnerability (whether social, familial, or psychological) corresponded to scores from 4 to 5 on the 5-point scale.

Data on parental experience in relation to the loss of the infant provided from direct question asked during the interview 3 months after infant death. They focused on their relationship with the team (feeling of consideration, clarity and precision of medical information), their satisfaction with end-of-life care (involvement in infant’s care, infant’s pain control, support from their relatives and from the referent psychologist, spiritual support by the hospital chaplain). We also asked if persistent disturbances of appetite and sleep had occurred following infant's death, and whether they could return to their employment. We finally questioned them about their feelings of guilt and anger and about prospects of a future pregnancy. Answers to these direct questions to parents were scored as binary (yes/no).

A document holder was given to the parents, containing information about the study, consent document, and, for each parent, two HADS questionnaires to be completed 5 months and 15 months after death, with two stamped envelopes in the name of the principal investigator.

#### HADS questionnaire

This screening tool, developed Zigmond and Snaith [13] and validated in its French version [14], includes 14 questions. Seven questions screen for anxiety (HADS-A) and seven screen for depression (HADS-D). Each question is rated from 0 to 3, giving sub-scores between 0 and 21, with cut-off value for each subscale > 8 to identify anxiety or depression [15].

### Statistical analysis

Comparisons were made using the Fisher, chi-squared, Student, and Wilcoxon-Mann–Whitney tests, as appropriate.

Based on a report from our department of an equivalent distribution of death rate occurring after a decision of WWLST or despite maximum care [9], and under the assumption of a 50% rate of parental anxiety or depression 5 months after the loss of their infant, 50 parents per group were needed to demonstrate a 20% difference in the rate of anxiety or depression in at least one of the parents according to death condition, with a power of 80% and *p* < 0.05. Given that the annual number of deaths in the department is about 40 and that the parental participation rate in the interview 3 months after infant death is about 60%, the recruitment period was set at 5 years.

Statistical analysis was performed with SAS Version 9 software (SAS Institute, Cary, NC). Values are expressed as numbers (%) or medians (Q25, Q75). *p* < 0.05 was considered statistically significant.

## Results

### Population

**Fig. 1:**
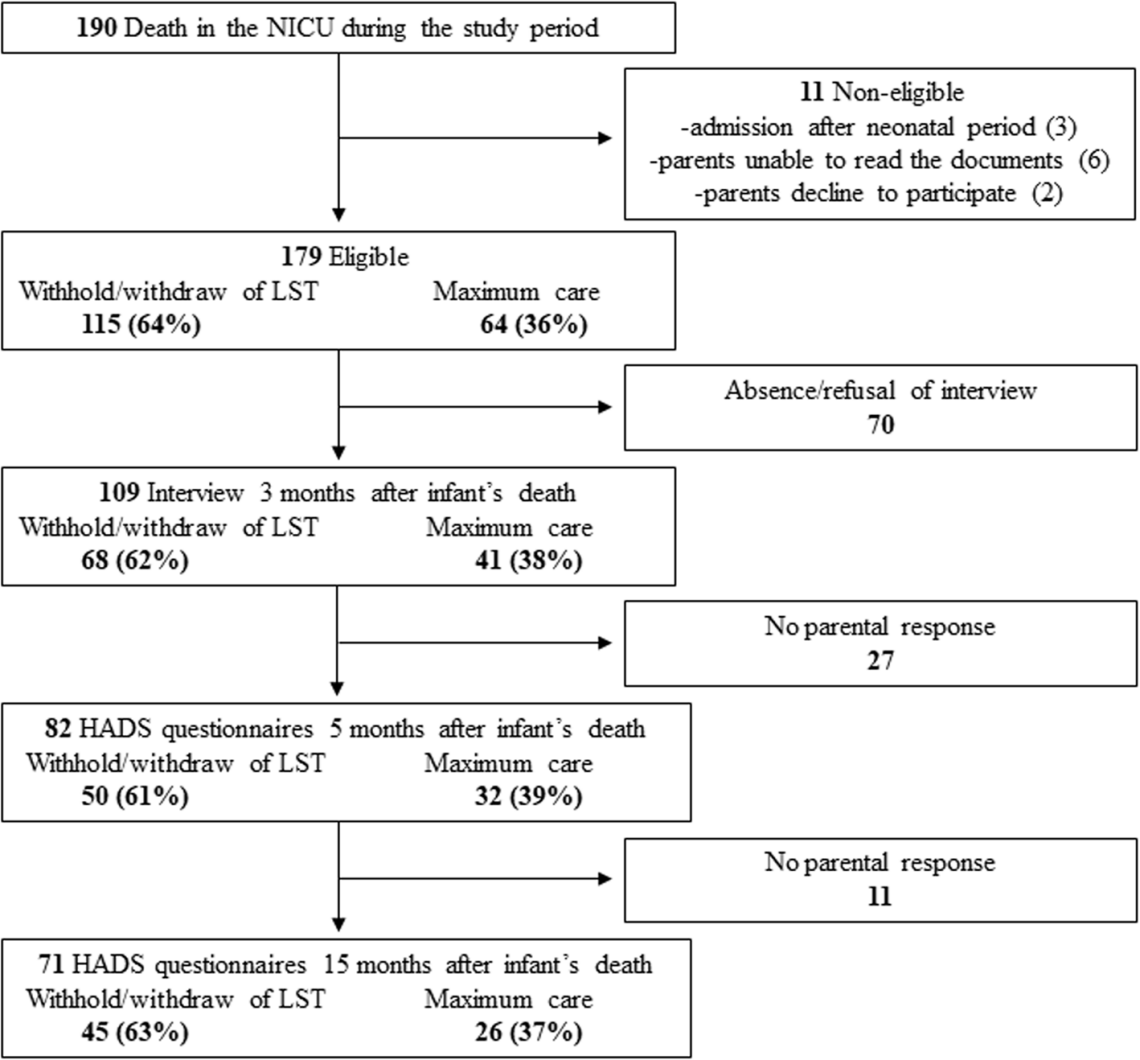
Flow chart of the study population. NICU, neonatal intensive care unit; LST, life-sustaining treatment; HADS, Hospital Anxiety and Depression Scale

One hundred and ninety infants died in the department of neonatal medicine during the study period. Among them, 179 were eligible, including 115 (64%) infants who died after a decision of WWLST and 64 (36%) despite maximum care (Fig. 1). Perinatal characteristics were comparable between groups (Table 1).

**Table 1:** Perinatal characteristics according to the context of death

	**Withhold/withdraw LST** (*N* = 115)	**Maximal care** (*N* = 64)	p
Maternal age (years)	30 (26, 35)	30 (26, 34)	0.77
Paternal age (years)	31 (27, 40)	31 (28, 39)	0.82
Separated parents	5 (4)	2 (3)	1.0
Parity^a^	2 (1, 3)	2 (1, 3)	0.64
Multiple pregnancy	19 (17)	13 (20)	0.66
Assisted reproductive technology	16 (14)	11 (17)	0.55
Previous perinatal bereavement	17 (15)	13 (20)	0.34
Antenatal pediatric consultation	60 (52)	37 (58)	0.47
Gestational age (weeks)	32 (25, 38)	29 (26, 35)	0.27
Birthweight (g)	1624 (790, 2890)	1200 (760, 2480)	0.30
5 min Apgar score	7 (4, 9)	6 (2, 8)	0.16
Male	64 (56)	40 (62)	0.37
IUGR	27 (23)	15 (23)	1.0
Complicated childbirth^b^	64 (56)	40 (62)	0.37
Congenital malformation	24 (21)	10 (16)	0.39

Values are numbers (%) or medians (Q25, Q75)

*Abbreviations*: *LST* Life-sustaining treatment, *IUGR* Intrauterine growth restriction

^a^Including the current pregnancy

^b^Defined as obstetrical maneuvers, urgent cesarean delivery, intubation or maternal resuscitation

#### Multidisciplinary ethics meetings

The decision of WWLST was made following a collegial procedure in all cases. The reasons for the meeting, the composition and the decisions, as well as the opinions of the parents are provided in Table 2.

**Table 2:** Main characteristics of multidisciplinary ethics meetings (*N* = 115)

**Reason**^**a**^	
Low quality of life expected	96 (83)
Disproportionate or unreasonable treatment	32 (28)
Ineffective treatment	22 (19)
**Composition**	
Physicians of the department	6 (4, 7)
Involved directly in the patient’s care	2 (1, 2)
Not involved directly in the patient’s care	3 (2, 5)
Residents	1 (0, 2)
Physicians not assigned to the department	1 (1, 2)
Palliative care specialist	69 (60)
Surgeon	20 (17)
Specialist in radiology and medical imaging	17 (15)
Specialist in medical genetics	14 (12)
Specialist in pediatric neurology	12 (10)
Specialist in neurophysiology	12 (10)
Family physician	1 (0.9)
Paramedical personnel	2 (1, 3)
Referent nurse of the infant	104 (90)
Nurse manager	53 (46)
Childcare assistant	7 (6)
Psychologist	106 (92)
**Decision**^**a**^	
Withholding of LST	90 (78)
Resuscitation	90 (78)
Use of catecholamines/vasopressors	36 (31)
Invasive mechanical ventilation	55 (48)
Surgery	16 (14)
Renal replacement therapy	3 (16)
Withdrawal of LST	58 (50)
Invasive mechanical ventilation	52 (45)
Catecholamines/vasopressors	9 (8)
Antibiotics	9 (8)
Parenteral or enteral feeding	2 (2)
**Parental agreement with the decision**^**b**^	
Explicit agreement	49 (43)
Tacit agreement	41 (36)
Absence of agreement	10 (9)
Impossible/difficult to adjudicate	15 (13)

Values are numbers (%) or medians (Q25, Q75)

*Abbreviations*: *LST* Life-sustaining treatments

^a^May be multiple for some infants

^b^According to the referent physician of the department for the infant, after parental interview following the ethics debate

#### Death

The infant's postnatal age at the time of death was earlier in the event of death despite maximum care compared to death after a WWLST decision (Table 3). Severe brain lesions and congenital anomalies accounted for nearly three quarters of the deaths after the decision of WWLST, while death despite maximum care occurred mainly in the context of respiratory failure, infection or extreme prematurity.

**Table 3:** Age, context, infant’s status and environment at time of death

	**Withhold/withdraw LST** (*N* = 115)	**Maximal care** (*N* = 64)	p
**Age** (days)	12 (8, 142)	3 (1, 140)	0.02
**Context**			< 0.001
Severe brain lesions	63 (55)	0 (0)	
Severe congenital malformation	28 (24)	2 (3)	
Respiratory failure	0 (0)	20 (31)	
Sepsis	0 (0)	13 (20)	
NEC, other GI disease	11 (10)	7 (11)	
Extreme immaturity	13 (11)	22 (34)	
**Presence with the infant**			
Mother	100 (87)	52 (81)	0.31
Father	83 (72)	45 (70)	0.79
Both parents	81 (70)	42 (66)	0.51
Siblings	2 (2)	1 (2)	1.0
Other family members	15 (13)	9 (14)	1.0
Absence of family members	5 (4)	7 (11)	0.17
Death in arms of			0.016
Mother	74 (64)	43 (67)	
Father	18 (16)	2 (3)	
Family members/caregivers	11 (10)	5 (8)	
Nobody	12 (10)	14 (22)	
**Perception of the infant's condition and environment by caregivers**^**a**^			
Satisfactory pain control	108 (94)	58 (91)	0.61
Satisfactory PS by relatives	104 (90)	50 (78)	0.04
Proposal of			
Psychological support	89 (77)	39 (61)	0.03
Spiritual support	78 (68)	30 (47)	0.01

Values are numbers (%) or medians (Q25, Q75)

*Abbreviations*: *LST* Life-sustaining treatments, *NEC* Necrotizing enterocolitis, *GI* Gastrointestinal, *PS* Parental support

^a^Joint opinion of the physician and nurse present at the time of death, satisfactory corresponds to scores from 4 to 5 on a 5-point scale

One hundred and fifty-seven infants (88%) died in the presence of at least one of their parents, with no difference between groups. The endotracheal tube was removed in 52 (45%) infants of the group with a decision of WWLST. Death in human arms and in father’s arms were more frequent after a decision of WWLST.

Caregivers present at the time of death felt that parents were better accompanied by relatives and were more often offered psychological and spiritual support in the event of WWLST (Table 3).

#### Interview 3 months after infant death

Of the 179 bereaved parents, 109 (61%) attended the 3-month interview. Among them, 68 (62%) had lost their infant after the decision of WWLST and 41 (38%) despite maximum care.

Data on family environment, collected since hospitalization, were comparable between the groups (Table 4).

**Table 4:** Data collected during interview with parents 3 months after infant's death

	**Withhold/withdraw LST** (*N* = 68)	**Maximal care** (*N* = 41)	p
**Assessment of family environment**			
Satisfactory^a^			
Language expression/understanding	59 (87)	31 (76)	0.14
Communication within the couple	47 (69)	26 (63)	0.54
Support from the relatives	49 (72)	27 (66)	0.49
Vulnerability^a^			
Social factors	15 (22)	9 (22)	0.99
Family factors	18 (26)	8 (20)	0.41
Individual, mother	19 (28)	8 (20)	0.32
Individual, father	7 (10)	5 (12)	1.0
**Parental experience in relation to the loss of the infant**^**b**^			
Relationship with the team			
Feeling of listening, consideration	59 (87)	31 (76)	0.14
Clarity of medical information	64 (94)	32 (78)	0.03
Detailed medical information	63 (93)	31 (76)	0.03
Satisfaction with			
Presence and involvement in care	61 (90)	31 (76)	0.049
Infant's pain control	45 (66)	19 (46)	0.04
Support by relatives	60 (88)	28 (68)	0.01
Proposal of psychological support	62 (91)	36 (88)	0.81
Proposal of spiritual support	38 (56)	11 (27)	0.006
Following infant’s death			
Persistent appetite disturbance	11 (16)	8 (20)	0.85
Persistent sleep disturbance	22 (32)	16 (39)	0.62
Return to work	57 (84)	31 (76)	0.42
Prospect of future pregnancy	41 (60)	14 (34)	0.01
Feelings^c^			
Guilt	23 (34)	17 (41)	0.55
Anger	26 (38)	17 (41)	0.74

Values are numbers (%)

^a^Joint opinion of the principal investigator and the referent psychologist. “Satisfactory” and “Vulnerability” corresponds to scores from 4 to 5 on a 5-point scale

^b^Binary responses to direct questions to parents. Numbers and percentages indicate positive answers

^c^Direct and spontaneous expression during the interview

Parents whose infants died after WWLST reported more frequently that they had received clear and detailed medical information, support from their relatives, and proposals of spiritual support by a hospital chaplain. In addition, they more often considered the prospect of a future pregnancy. Their involvement in care was higher, and their perception of their infant's pain control at the time of death was better. Appetite and sleep disturbances, feelings of guilt and anger, and return to work were comparable between groups (Table 4). In the cohort as a whole and compared to fathers, mothers had more frequent disturbances in appetite (16.5% vs. 2.7%, *p* < 0.001) and sleep (28% vs. 15%, *p* = 0.02). They also more often felt guilt (37% vs. 4.5%, *p* < 0.001).

### Parental anxiety and depression 5 months and 15 months after infant death

Of the 109 couples interviewed 3 months after death, 140 HADS questionnaires were completed by 70 couples, 11 by mothers only and 1 by the father only, ie, in total 152 HADS questionnaires from 82 couples, including 81 completed by mothers, and 71 by fathers. At 15 months, 110 HADS questionnaires were completed by 55 couples, 15 by mothers only and 1 by the father only, ie, in total 126 HADS questionnaires from 71 couples, including 70 completed by mothers, and 56 by fathers.

**Fig. 2:**
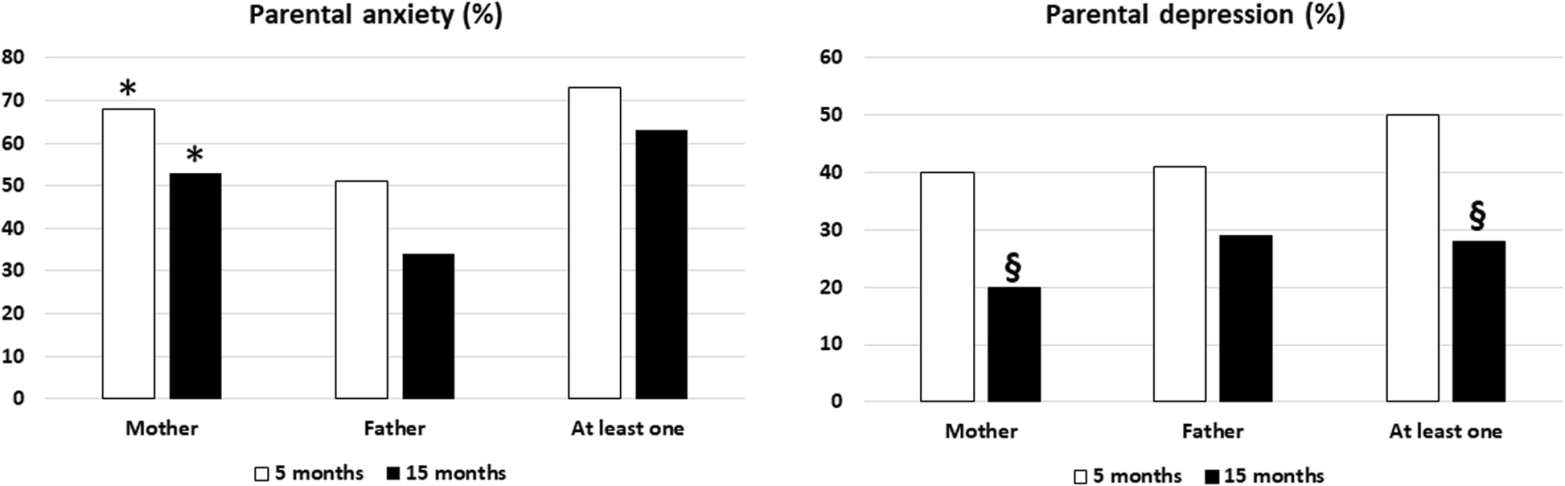
Rates of parental anxiety and depression 5 months and 15 months after infant’s death, according to the Hospital Anxiety and Depression Scale (HADS).\ *p < 0.05 vs. fathers, §*p* < 0.01 vs. 5 months

Five months after death, HADS scores were consistent with anxiety in at least one parent in 73% of the cases and with depression in 50%. At 15 months, these rates were, respectively, 63% (*p* = 0.19 vs. 5 months) and 28% (*p* = 0.006 vs. 5 months) (Fig. 2).

#### Relationship to parental gender

Anxiety rates were higher in mothers than in fathers at 5 months (68% vs. 51%, *p* = 0.031) and 15 months (53% vs. 34%, *p* = 0.034). Between these two time-points, the change in anxiety rates was not significant in either mothers (*p* = 0.06) or fathers (*p* = 0.06) (Fig. 2).

Depression rates were comparable between mothers and fathers at 5 months (40% vs. 41%, *p* = 0.87) and 15 months (20% vs. 29%, *p* = 0.26). Between these two time-points, the change in depression rates was significant for mothers (*p* = 0.009) but not fathers (*p* = 0.15) (Fig. 2).

#### Relationship to the circumstance of death

No association was found between the circumstance of death and parental anxiety at 5 months and 15 months.

The risk of depression at 5 months in at least one of the parents (OR 0.35, 95% CI 0.14–0.88, *p* = 0.02) and in the fathers (OR 0.32, 95% CI 0.12–0.86, *p* = 0.02) was lower in the event of death after WWLST (Table 5).

**Table 5:** Parental anxiety and depression 5 months and 15 months after infant’s death

	**Withhold/withdraw LST**	**Maximal care**	**p**
**Parental anxiety**^**a**^			
5 months			
Mothers (*n* = 81)	32/50 (64)	23/31 (74)	0.34
Fathers (*n* = 71)	19/41 (46)	17/30 (57)	0.39
At least one of the parents (*n* = 82)	35/50 (70)	25/32 (78)	0.42
15 months			
Mothers (*n* = 70)	24/45 (53)	13/25 (52)	0.91
Fathers (*n* = 56)	9/35 (26)	10/21 (48)	0.09
At least one of the parents (*n* = 71)	27/45 (60)	18/26 (69)	0.44
**Parental depression**^**b**^			
5 months			
Mothers (*n* = 81)	17/50 (34)	15/31 (48)	0.20
Fathers (*n* = 71)	12/41 (29)	17/30 (57)	0.02
At least one of the parents (*n* = 82)	20/50 (40)	21/32 (66)	0.02
15 months			
Mothers (*n* = 70)	10/45 (22)	4/25 (16)	0.76
Fathers (*n* = 56)	10/35 (29)	6/21 (29)	1.0
At least one of the parents (*n* = 71)	12/45 (27)	8/26 (31)	0.71

Values are numbers (%)

^a^Defined as a score in the sub-scale anxiety (HADS-A) of the Hospital Anxiety and Depression Scale (HADS) > the threshold value of 8

^b^Defined as a score in the sub-scale depression (HADS-D) of the Hospital Anxiety and Depression Scale (HADS) > the threshold value of 8

#### Relationship to parental agreement with the decision of WWLST

Explicit parental agreement after the multidisciplinary ethics meeting was associated with higher risks of anxiety at 5 months (OR 16.6, 95% CI 2.0–140.6, *p* = 0.005). No association was found with parental anxiety or depression at 5 months and 15 months in cases of explicit agreement with the decision of WWLST expressed 3 months after death (Table 6).

**Table 6:** Agreement with the decision of withholding/withdrawing life-sustaining treatment and parental anxiety and depression 5 months and 15 months after the infant death

	**Explicit**	**Other**^**a**^	p
**Parental anxiety**^**b**^			
5 months			
Agreement after ethics debate	19/20 (95)	16/30 (53)	0.005
Agreement 3 months after death	15/21 (71)	20/29 (69)	1
15 months			
Agreement after ethics debate	14/18 (78)	13/27 (48)	0.09
Agreement 3 months after death	14/21 (67)	13/24 (54)	0.58
**Parental depression**^**c**^			
5 months			
Agreement after ethics debate	11/20 (55)	9/30 (30)	0.14
Agreement 3 months after death	11/21 (52)	9/29 (31)	0.22
15 months			
Agreement after ethics debate	8/18 (44)	4/27 (15)	0.06
Agreement 3 months after death	7/21 (33)	5/24 (21)	0.34

Values are numbers (%)

^a^Include tacit agreement, absence of agreement, and impossible/difficult to adjudicate

^b^At least one parental score on the anxiety subscale (HADS-A) of the Hospital Anxiety and Depression Scale (HADS) > the threshold value of 8

^c^At least one parental score on the depression subscale (HADS-D) of the Hospital Anxiety and Depression Scale (HADS) > the threshold value of 8

## Discussion

This study showed that approximately 2/3 of neonatal deaths occurred in a context of WWLST, and 1/3 despite maximum care. Context of neonatal death influenced parental perception of end-of-life care and received support by professionals and relatives, as the risk of parental anxiety or depression in the first year of bereavement.

Our data are consistent with other single-center, regional and network studies, which found that a majority of newborn deaths occurred in context of WWLST [8, 16–19]. As noted, the two main causes in this context were severe brain damage and severe congenital malformations [18, 20]. As often reported in the neonatal period, decision-making mainly centered on quality of life considerations and, in much lower proportions, on the lack of further treatment benefit or ineffectiveness of care [17, 18, 21]. Death despite maximum care occurred in the context of acute respiratory failure or multi-organ failure, including sepsis and extreme prematurity [20]. The age of death was significantly earlier in this context, which highlights the somatic vulnerability of these infants.

Parental satisfaction with NICU staff is underpinned by the accuracy of the information provided, as well as calm, confident and controlled communication [22]. In the context of a death in the NICU, parents also stress the importance of healthcare providers’ guidance for bonding with their infant and creating memories [23]. The most frequently expressed regret is not to have spent enough time with their baby [24]. These observations provide insight into some of the differences between the two groups related to the parents’ relationship with the team and their satisfaction with their infant’s care. More parents in the context of WWLST reported satisfactory control of their infant's pain, but rates were low in both groups and significantly lower than the caregivers' estimates [25, 26]. Among bereaved parents, the perception of infant suffering at the end of life in the NICU has been associated with the decision to have another child [27]. We observed that prospect of a future pregnancy was indeed more frequent in the event of WWLST.

The physician and nurse present at the time of death also perceived a more optimal environment for the parents in the context of WWLST. Caregivers generally encouraged physical contact with the infant at the time of death. Nevertheless, about 15% of the deaths occurred in the incubator. For personal or cultural reasons, some parents or family members may feel discomfort with physical and emotional closeness to the infant at this time [25]. Context also plays a role since death in the incubator was two times less frequent in the event of WWLST. Removal of the endotracheal tube, practiced in nearly half the patients in this group, may favor the infant’s placement in the arms of parents for the last moments. As highlighted by Wilkinson et al., in infants going to die despite treatment, transition to palliative care is now the standard of care [16].

The high rates of anxiety and depression observed in bereaved parents during the first 15 months after the infant’s death is consistent with the literature, and about two–three times the rates among community parents [28–33]. Less intense grief reactions in fathers have generally been reported and were explained partly by their expected role of “supportive partner” [28, 29, 32, 34, 35]. Our results did not fully confirm these gender differences in mental health: rates were indeed higher for anxiety in mothers, but comparable for depression, and the lower rate of depression over time was mainly driven by the mothers. Several recent reviews have pointed out that the psychological and emotional impact of neonatal death in men, which has been under-explored compared to women, varied considerably between studies [34, 36].

In this cohort, death after a decision of WWLST was not associated with an increased risk of parental anger, guilt, anxiety or depression. Indeed, most parents with an infant hospitalized in an intensive care unit can imagine situations in which they would consider withdrawing LST, notably suffering, ineffective therapies, and quality of life considerations [37]. The differences observed for parental mental health indicators according to the context of the death mainly raise questions about family well-being after infant death despite maximum care. An infant’s rapid deterioration and the ineffectiveness of the medical acts performed can generate the feeling, among caregivers and parents, of a total lack of control in the clinical history, including the final moment of the infant’s death. Previous studies have pointed to the psychological benefits of taking control and self-efficacy and the risk of parental depression following rapid or sudden child loss [38, 39].

The French law specifies that parents’ opinion of the medical decision of WWLST for their child must be obtained [10]. In practice, few data accurately address parental involvement in the decision-making process, in NICUs [40] as in pediatric intensive care units [41]. These studies highlight the gap between the literature, which emphasizes that parents generally prefer to be involved in the decision, and the difficulty of reaching a genuinely shared decision in daily practice [42, 43]. The question we asked the parents, namely their degree of approval of a decision already made by the medical team, reflects the preferential choice of our team that parents participate in the decision without being directly responsible for it, which appears as intermediate between a paternalistic approach and a shared decision [40]. In reality, however, some parents during the 3-month interview reported having been the main actors in the decision-making process, using the words “we were asked to decide.” A recent qualitative study underlined that most parents could “well live” with the decision they made for their child [44]. The results of our work also suggest that the parental perception of having explicitly agreed to the decision, or even of having been an actor in the decision, can change over time. This dynamic is illustrated in the variable association with parental anxiety at 5 months, higher when expressed during hospitalization but not when expressed 3 months later.

The results of this single-center study may be difficult to extrapolate to other centers. Death after a decision of WWLST depended on the essential condition that provision of care appeared as "unreasonable obstinacy". However, this condition was not sufficient in itself, as we definitely classified a death in this group if, in this condition, a multidisciplinary ethics meeting was also held. Death despite maximum care sometimes occurred too early to allow for the organization of the meeting, and it cannot be excluded that, if longer survival had been possible, some of these situations would have led to decisions of WWLST. Maximum care for a patient may also be defined differently, depending on the center. For example, in the case of congenital diaphragmatic hernia, death without recourse to extracorporeal membrane oxygenation may indicate that maximum care has been given for one center and yet be defined as withholding care for another [45].

Another weakness is our limited number of patients. Our calculation of the number of subjects needed was based on an equivalent distribution of patients according to the context of death from a study carried out in the department in the 2000s [9]. However, as observed in other centers, death in a context of WWLST has now become the majority condition [17–21, 46]. The participation rate in the interview 3 months after infant death was consistent with our prevision and with the rates usually reported in studies on this topic [47–49]. In addition, context of death had no influence on attendance of the meeting, with the distribution between the two groups at 3 months being very close to that observed during hospitalization. Our sample size was sufficient to observe a significant difference between the groups for the primary endpoint, i.e., the rate of anxiety or depression in at least one of the parents at 5 months. In this respect, it is also important to note that rates of parental anxiety and depression were probably affected by the consultation carried out 3 months after infant death, which allowed them to obtain information, particularly on the cause of the death, to receive emotional support, and to express their experiences with their infant and the caregivers. A recent review showed that these conversations are beneficial for a large majority of parents [50].

## Conclusions

This study shows that the context of death has a significant and prolonged impact on the emotional experience of bereaved parents. It highlights the need for training of healthcare professionals to provide effective support to bereaved parents [51]. Notably, a medico-psychological appointment should be systematically offered to parents to discuss all events surrounding their infant’s death, and to welcome their feedback on the care given. This can facilitate meaning-making processes and, potentially, the acceptance of death [52].

## Supplementary Information

**Additional file 1.**

## Data Availability

The datasets used and /or analysed during the current study are available from the corresponding author on reasonable request.

## Abbreviations

HADS: Hospital Anxiety and Depression Scale
NICU: Neonatal intensive care unit
WWLST: Withholding or withdrawing life-sustaining treatment
