# Supplementary material for: Context of a neonatal death affects parental perception of end-of-life care, anxiety and depression in the first year of bereavement
Source: BMC Palliat Care. 2023 May 13;22:58. doi: 10.1186/s12904-023-01183-8 (PMC10182590; doi:10.1186/s12904-023-01183-8)
Supplement: Supplementary file 1 — Additional file 1. [file 12904_2023_1183_MOESM1_ESM.pdf]

Experience of parents after death of their infant in the neonatal period

CASE REPORT FORM

PATIENT N° : _ _ _
INITIALS

SURNAME :|__|__|__| FIRST NAME :|__|__|

Initials |__|__|__| |__|__| Patient number |__|__|__|

Infant initials |__|__|__| |__|__|
(first 3 letters of surname, first 2 letters of first name)

Birthdate |__|__| |__|__| |__|__|__|__|

Sex: Male ☐ Female ☐

- **INCLUSION CRITERIA**

Infant deceased in the department of neonatal medicine:

Yes ☐ No ☐

Written informed consent from both parents:

Yes ☐ No ☐

If a 'NO' answer is checked, the patient is not included in the study

- **NON INCLUSION CRITERIA**

Age >28 days at admission, if born full term OR >41 weeks of corrected gestational age, if born preterm:

Yes ☐ No ☐

Decline to participate of at least one of the parents :

Yes ☐ No ☐

Parents cannot read French:

Yes ☐ No ☐

If a 'YES' answer is checked, the patient is not included in the study

Patient included in the study?

Yes ☐ No ☐

If yes

Patient number |__|__|__|

Initials |__|__|__| |__|__| Patient number |__|__|__|

- **PERINATAL HISTORY**

Mother's age |__|__| years

Mother's occupation:

Father's age |__|__| years

Father's occupation:

Medical background:

Mother:

Father:

Pregnancy:

Number of previous gestations |__|__| Parity |__|__|

Obstetric history:

Assisted reproductive technology: Yes □ No □

Previous perinatal bereavement: Yes □ No □

Antenatal interview of parents with a pediatrician:

Yes □ No □

Parental separation during pregnancy:

Yes □ No □

Birth:

Gestational age |__|__| weeks |__|__| days Birthweight |__|__|__|__| grams

IUGR (**Definition**): Yes □ No □

Complicated childbirth (**Definition**): Yes □ No □

5 minutes Apgar score |__|__|

Congenital malformation: Yes □ No □

Multiple pregnancy: Yes □ No □

IUGR: Intrauterine growth restriction: birthweight ≤-2DS according to Usher and Mac Lean.
Complicated childbirth: Defined as obstetrical maneuvers, urgent cesarean delivery, intubation or maternal resuscitation.

Initials |__|__|__| |__|__| Patient number |__|__|__|

- **MULTIDISCIPLINARY ETHICS MEETING**

Multidisciplinary ethics meeting Yes □ No □

If yes:

Date of the meeting |__|__| |__|__| |__|__|__|__|

Duration: start |__|__| hour |__|__| min end |__|__| hour |__|__| min

Meeting requested by the referent physician:

Yes □ No □ Other □

If other, precise:

Somatic features:

Post-natal age |__|__| days Corrected GA |__|__| weeks Weight |__|__|__|__| gr

Respiratory support:

None □ Noninvasive ventilation □ Conventionnal ventilation □

High frequency oscillatory ventilation □ Inhaled NO □ FiO_2 |__|__| %

Neurologic assessment (multiple choice possible):

IVH (**Definition**): Yes □ No □

Right maximum grade |__| Left maximum grade |__|

PVL (**Definition**): Yes □ No □

Right maximum grade |__| Left maximum grade |__|

Hypoxic-ischemic encephalopathy: Yes □ No □

Clinical grade (Sarnat) (**Definition**) |__| EEG grade (**Definition**) |__|

MRI cerebral lesions (multiple choice possible):

Cortical □ Subcortical □ Basal ganglia □

White matter □ Cerebellum □ Brainstem □

Intraventricular hemorrhage (IVH) grades: 1 = Subependymal hemorrhage; 2 = Moderate intraventricular hemorrhage (<50% of lateral ventricular surface); 3 = Intraventricular hemorrhage (>50% of lateral ventricular surface) with ventricular dilation; 4 = Intraventricular hemorrhage with ischemohemorrhagic infarction.
Periventricular leukomalacia (PVL) grades: 1 = Localized periventricular hyperechogenicity; 2 = Extensive periventricular hyperechogenicity; 3 = Small cavities (< 5 mm); 4 = Large cavities (> 5 mm).
Sarnat's classification: 1. Mild = various disturbances of tone with hyperexcitability without disturbance of consciousness and primary reflexes; 2. Moderate = disorders of tone, consciousness, primary reflexes and possibility of convulsions; 3. Severe = lethargic or comatose state and/or status epilepticus.
EEG classification: 1. Normal or subnormal (rapidly hyperactive); 2. Intermediate = abnormal EEG in the first 48 hours but normalization on Day 7 or isolated seizures during < 48 hours; 3. Poor = lack of organization at 48 hours or prolonged discontinuous pattern (A >7 days, B >48 hours) or isolated convulsions lasting >48 hours or status epilepticus <48 hours; 4. Very poor = inactive or paroxysmal or poor tracings + theta for more than 12 hours or discontinuous type B tracing >7 days or status epilepticus >48 hours.

Hemodynamics (multiple choice possible):

Vasopressor	☐	Multiple transfusions (RBC, platelets)	☐
Renal replacement therapy	☐	Indication for ECMO	☐

Analgesic and sedative:

Midazolam |__|__|__|__| microg/kg/hr Sufentanil |__|__,|__|__| microg/kg/hr

Other drugs:

Name: Dosage:

Name: Dosage:

Name: Dosage:

Main failures (multiple choice possible):

Neurologic	☐	Cardiovascular	☐	Respiratory	☐
Renal	☐	Gastro-intestinal	☐	Other	☐

If other, precise:

Composition:

Physicians of the department:

Referent physisican	Yes	☐	No	☐

Others physisians involved in patient's care (number) |__|__|

Others physisians not involved in patient's care (number) |__|__|

Number of residents |__|__|

Staff members:

Referent psychologist	Yes	☐	No	☐
Nurse manager	Yes	☐	No	☐
Referent nurse	Yes	☐	No	☐
Childcare assistant	Yes	☐	No	☐

Physicians not assigned to the department:

Palliative care specialist	Yes	☐	No	☐
Surgeon	Yes	☐	No	☐
Specialist in radiology and medical imaging	Yes	☐	No	☐
Specialist in medical genetics	Yes	☐	No	☐
Specialist in pediatric neurology	Yes	☐	No	☐
Specialist in neurophysiology	Yes	☐	No	☐
Family physician	Yes	☐	No	☐
Other	Yes	☐	No	☐

If other, precise:

Main ethical issues (multiple choice possible):

Ineffective treatment	Yes	☐	No	☐
Low quality of life expected (**POPC**)	Yes	☐	No	☐
Disproportionate or unreasonable treatment (**Definition**)	Yes	☐	No	☐

Décision (multiple choice possible):

No decision: lack of consensus	Yes	☐	No	☐
Continuation of life-sustaining treatments	Yes	☐	No	☐
Withholding of life-sustaining treatments	Yes	☐	No	☐
Withdrawing of life-sustaining treatments	Yes	☐	No	☐

Life-sustaining treatments withheld (multiple choice possible):

Resuscitation	Yes	☐	No	☐
Use of catecholamines/vasopressors	Yes	☐	No	☐
Invasive mechanical ventilation	Yes	☐	No	☐
Specify the limitation:				
Surgery	Yes	☐	No	☐
Specify the limitation:				
Renal replacement therapy	Yes	☐	No	☐
Other	Yes	☐	No	☐
Specify the limitation:				

Simplified POPC (Pediatric Overall Performance Category) score: 1= Good overall performance: normal psychomotor development and schooling for age, normal activity in everyday life; 2 = Mild global impairment: able to follow normal schooling but at a lower than average level, minimal neurological deficit compatible with a normal and independent life; 3 = Moderate global deficiency: need for adapted schooling and/or learning deficit, moderate deficiency of an organ requiring monitoring and possibly limiting performance; 4 = Severe global impairment: dependent on others for daily activities, severe impairment of an organ making the child dependent on daily care; 5 = Coma or vegetative state: more or less profound unconsciousness without criteria of brain death, very limited interaction with the environment; 6 = Brain death.

Unreasonable treatment (Law n ° 2005-370 of April 22, 2005 relating to the rights of patients and the end of life): Implementation or maintenance of useless and disproportionate treatments, which have no other effect than the sole artificial maintenance of life.

Life-sustaining treatments withdrawn (multiple choice possible):

Use of catecholamines/vasopressors	Yes	☐	No	☐
Invasive mechanical ventilation	Yes	☐	No	☐
Antibiotics	Yes	☐	No	☐
Parenteral nutrition	Yes	☐	No	☐
Enteral feeding	Yes	☐	No	☐
Other	Yes	☐	No	☐

Specify the withdrawal:

Parental information and expression:

Information on the critical situation and ethical issues

Yes ☐ No ☐

Information on organization of a multidisciplinary ethics meeting

Yes ☐ No ☐

Parental expression on withholding/withdrawing life-sustaining treatment before the meeting

Yes ☐ No ☐

If yes, precise:

Information on the decision of the multidisciplinary ethics comitee

Yes ☐ No ☐

If yes, delay following the meeeting |__|__| hour |__|__| min

Opinion on the decision

Explicit agreement	☐	Tacit agreement (**Definition**)	☐
Absence of agreement	☐	Impossible/difficult to adjudicate	☐

Tacit agreement: Absence of explicit agreement, but parents whose attitude and/or expression suggest that they do not oppose the decision.

Initials |__|__|__| |__|__| Patient number |__|__|__|

- **INFANT DEATH**

Date of death |__|__| |__|__| |__|__|__|__|

Context:

Death despite maximal care Yes ☐ No ☐

Death after decision of withholding or withdrawing life-sustaining treatment

Yes ☐ No ☐

If yes, delay between ethics meeting and death |__|__| day |__|__| hour

Main cause of death among:

Severe cerebral lesions ☐

Severe congenital malformation ☐

Respiratory failure ☐

Sepsis, hemodynamic failure ☐

Necrotising enterocolitis, other severe gastrointestinal disease ☐

Extreme immaturity, multiple organ failure ☐

Other ☐

If other, precise:

Analgesic and sedative:

Sufentanil or other opioid |__|__,|__|__| micrograms/kg/hour

Midazolam or other sedative |__|__|__|__| micrograms/kg/hour

Other drugs:

Name: Dosage:

Name: Dosage:

Name: Dosage:

Environment:

Presence with the infant at the moment of death (multiple choice possible):

Mother Yes □ No □ Father Yes □ No □

Siblings Yes □ No □ Other family members Yes □ No □

Death in arms of:

Mother Yes □ No □ Father Yes □ No □

Other family members Yes □ No □ Caregivers Yes □ No □

Nobody (ie, death in the incubator) Yes □ No □

Perception of the infant's condition and environment by caregivers:

Satisfactory pain control/comfort (**Definition**) Yes □ No □

Satisfactory parental support by relatives (**Definition**) Yes □ No □

Proposal of psychological support Yes □ No □

Proposal of spiritual support Yes □ No □

Comments:

Pain control and parental support: Joint opinion of the physician and nurse present at the time of death, satisfactory corresponds to scores from 4 to 5 on a 5-point scale. 1 = very poor; 2 = somewhat poor; 3 = intermediate; 4 = fairly good; 4 = very good.

Initials |__|__|__| |__|__| Patient number |__|__|__|

- **INTERVIEW 3 MONTHS AFTER INFANT DEATH**

Parents came to the interview: Yes ☐ No ☐

If not, reason possibly given:

Date of the interview |__|__| |__|__| |__|__|__|__|

Parental information and opinion on the decision of the multidisciplinary ethics comitee:

Multidisciplinary ethics meeting Yes ☐ No ☐

If yes:

Information on organization of a multidisciplinary ethics meeting

Yes ☐ No ☐

Opinion on the decision

Explicit agreement	☐	Tacit agreement (**Definition**)	☐
Absence of agreement	☐	Impossible/difficult to adjudicate	☐

Parental experience in relation to the loss of the infant (Definition):

Relationship with the team:

Feeling of listening, consideration	Yes	☐	No	☐
Clarity of medical information	Yes	☐	No	☐
Detailed medical information	Yes	☐	No	☐

Satisfaction with:

Presence and involvement in care	Yes	☐	No	☐
Infant's pain control/comfort	Yes	☐	No	☐
Support by relatives	Yes	☐	No	☐
Proposal of psychological support	Yes	☐	No	☐
Proposal of spiritual support	Yes	☐	No	☐

Tacit agreement: Absence of explicit agreement, but parents whose attitude and/or expression suggest that they do not oppose the decision.

Parental experience in relation to the loss of the infant: Binary responses to direct questions to parents. If ambiguous answer, satisfactory corresponds to scores from 4 to 5 on a 5-point scale. 1 = very poor; 2 = somewhat poor; 3 = intermediate; 4 = fairly good; 4 = very good.

Following infant's death (Definition):

Persistent appetite disturbance:				
Mother	Yes	□	No	□
Father	Yes	□	No	□
Persistent sleep disturbance:				
Mother	Yes	□	No	□
Father	Yes	□	No	□
Return to work after legal maternity/paternity leave:				
Mother	Yes	□	No	□
Father	Yes	□	No	□
Feelings of guilt:				
Mother	Yes	□	No	□
Father	Yes	□	No	□
Feelings of anger:				
Mother	Yes	□	No	□
Father	Yes	□	No	□
Prospect of future pregnancy:	Yes	□	No	□

Comments:

Definition: Binary responses to direct questions to parents, except for guilt and anger (direct question only in absence of spontaneous expression during the interview).

Assessment of family environment:

Parental separation:	Yes	☐	No	☐

Satisfactory (Definition):

Langage expression/understanding	Yes	☐	No	☐
Communication within the couple	Yes	☐	No	☐
Support from the relatives	Yes	☐	No	☐

Vulnerability (Definition):

Social factors (financial difficulties for the everyday life):

Yes ☐ No ☐

Family factors (relationships and support from their own parents and siblings):

Yes ☐ No ☐

Individual, mother (past-history of bereavement or requirement of psychotropic medication):

Yes ☐ No ☐

Individual, father (past-history of bereavement or requirement of psychotropic medication):

Yes ☐ No ☐

Comments:

Document holder (parental information and consent and HADS questionnaires) given to parents:

Yes ☐ No ☐

Satisfactory: Joint opinion of the principal investigator and the referent psychologist. Corresponds to scores from 4 to 5 on a 5-point scale. 1 = very poor; 2 = somewhat poor; 3 = intermediate; 4 = fairly good; 4 = very good.

Vulnerability: Joint opinion of the principal investigator and the referent psychologist. Corresponds to scores from 4 to 5 on a 5-point scale. 1= not at all; 2 = probably not; 3 = uncertain; 4 = present; 5= very present.

Initials |__|__|__| |__|__| Patient number |__|__|__|

- **FIVE MONTHS AFTER INFANT DEATH**

Consent document received: Yes ☐ No ☐

HADS score:

Mother:

HADS questionnaire received Yes ☐ No ☐

If yes, date |__|__| |__|__| |__|__|__|__|

HADS score

HADS Anxiety |__|__|

Significant anxiety (**Definition**) Yes ☐ No ☐

HADS Depression |__|__|

Significant depression (**Definition**) Yes ☐ No ☐

Father:

HADS questionnaire received Yes ☐ No ☐

If yes, date |__|__| |__|__| |__|__|__|__|

HADS score

HADS Anxiety |__|__|

Significant anxiety (**Definition**) Yes ☐ No ☐

HADS Depression |__|__|

Significant anxiety (**Definition**) Yes ☐ No ☐

Personalized follow-up by the referent psychologist or organization of a local follow-up:

Mother: Yes ☐ No ☐

If yes, reason:

Father: Yes ☐ No ☐

If yes, reason:

Significant anxiety or depression: Defined as a score in the sub-scale anxiety (HADS-A) or in the sub-scale depression (HADS-D) >8.

Initials |__|__|__| |__|__| Patient number |__|__|__|

- **FIFTEEN MONTHS AFTER INFANT DEATH**

HADS score:

Mother:

HADS questionnaire received Yes ☐ No ☐

If yes, date |__|__| |__|__| |__|__|__|__|

HADS score

HADS Anxiety |__|__|

Significant anxiety (**Definition**): Yes ☐ No ☐

HADS Depression |__|__|

Significant depression (**Definition**): Yes ☐ No ☐

Father:

HADS questionnaire received Yes ☐ No ☐

If yes, date |__|__| |__|__| |__|__|__|__|

HADS score

HADS Anxiety |__|__|

Significant anxiety (**Definition**): Yes ☐ No ☐

HADS Depression |__|__|

Significant anxiety (**Definition**): Yes ☐ No ☐

Personalized follow-up by the referent psychologist or organization of a local follow-up:

Mother: Yes ☐ No ☐

If yes, reason:

Father: Yes ☐ No ☐

If yes, reason:

Significant anxiety or depression: Defined as a score in the sub-scale anxiety (HADS-A) or in the sub-scale depression (HADS-D) >8.

Initials |__|__|__| |__|__| Patient number |__|__|__|

- **END OF STUDY**

End of study according to protocol:

	Yes	☐	No	☐
	If no, study exit date	|__|__| |__|__| |__|__|__|__|		

If premature end of study, reason:

Withdrawal of consent

Form mother :	Yes	☐	No	☐
Form father :	Yes	☐	No	☐
Other reason	Yes	☐	No	☐

If yes, precise:

- **ATTESTATION**

I, the undersigned, Doctor

certify that the data collected in this case report form are real and accurate.

Date |__|__| |__|__| |__|__|__|__|

Signature
